# Comparative Responses of Silicon to Reduce Cadmium and Enrich Selenium in Rice Varieties

**DOI:** 10.3390/foods12081656

**Published:** 2023-04-15

**Authors:** Yang Su, Xin Huang, Ling Li, Zahir Ahsan Muhammad, Meilin Li, Tengda Zheng, Zhe Guo, Yue Zhang, Dan Luo, Xiaoying Ye, Xiaomei Jia, Faiz Hussain Panhwar, Myo Thuzar Tun, Jianqing Zhu

**Affiliations:** 1 Rice Research Institute, Sichuan Agricultural University, 211, Huimin Road, Wenjiang District, Chengdu 611130, China; 2 Demonstration Base for International Science & Technology Cooperation of Sichuan Province, 211, Huimin Road, Wenjiang District, Chengdu 611130, China

**Keywords:** silicon fertilizer, cadmium pollution, bio-fortification, translocation, Se-enriched rice

## Abstract

Cadmium (Cd), a highly toxic heavy metal for crops in China, poses a significant threat to rice cultivation. It is crucial to identify the genotypes with robust resistance to heavy metals, including Cd, in rice. The experiment was conducted to examine the mitigation effect of silicon (Si) on Cd toxicity levels in Se-enriched Z3055B and non-Se-enriched G46B rice genotypes. A basal dose of Si improved the growth and the quality of rice significantly by reducing the Cd content in rice roots, stems, leaves and grains and increased the yield, biomass and selenium (Se) content of brown rice in both genotypes. Additionally, Se content in brown rice and polished rice was notably higher in Se-enriched rice than in non-Se-enriched rice, with the highest amount at 0.129 mg/kg and 0.085 mg/kg, respectively. The results demonstrated that a basal fertilizer concentration of 30 mg/kg of Si was more effective in reducing Cd transport from roots to shoots in Se-enriched rice than in non-Se-enriched rice genotypes. Therefore, it can be concluded that Se-enriched rice genotypes are a viable option for food crop production in Cd-contaminated areas.

## 1. Introduction

Cadmium (Cd) is one of the most widely prevalent and hazardous heavy metals in the soil, exerting lethal effects on crop life processes [1]. Accumulation of cadmium (Cd) in rice plants has been found to have a significant deleterious effect on various aspects of plant physiology and biochemistry, including photosynthesis, biomass production, and antioxidant enzyme activity [2]. It has both natural and anthropogenic sources, transferred to the crops via the soil, water and atmosphere, resulting in the accumulation of Cd in edible portions of the crop to threaten human health [3]. Research has demonstrated that, due to its biological half-life of 10 to 30 years [4], the majority of cadmium that has accumulated in the liver and kidneys of the human organism will persist even after exposure to the element has ceased [5]. The recent joint report from China’s Ministry of Environmental Protection (MEP) and Ministry of Land and Resources (MLR) indicates that cadmium was the most prevalent heavy metal pollutant, exceeding the MEP limit to a significant degree [6]. For example, the soil Cd pollution was as high as 5 times over the limit in Hunan Province [7]. The extent of farmland contaminated with by cadmium in China has reached 2.0 × 10^7^ hm^2^, comprising approximately one-fifth of the total farmland area in the country, with an estimated annual loss of 20 billion yuan [8]. Contaminated soil with high levels of cadmium can cause health risks by entering the food chain through rice, which is a staple food for half of the world’s population [9]. As such, the pollution of farmland by heavy metals is an urgent environmental issue that has gained global attention [10,11].

Silicon (Si), the most prevalent element in the earth’s crust after oxygen (O), is generally regarded as beneficial nutrient for the growth of plants [12,13]. Numerous evidences demonstrated that Si has a positive impact on the resistance to biological stresses such as brown spot disease, rice plant hopper, and sheath blight [14], and abiotic stresses such as heavy metals [15] during the growth and development of crops. The absorption of heavy metals in plants and the transportation of heavy metals from the soil to the roots were reduced by silicon (Si), while the absorption of mineral elements by plants was enhanced and the antioxidant capacity of plant cells was improved, which provided a protective mechanism against cytotoxicity [16]. Multiple studies reported that the toxicity effects of Cd in rice [17] and cabbage [18] were reduced by Cd-Si complex deposition in the cell wall. Research in this field primarily focused on the application Si to detoxify Cd in vegetables and ordinary grain crops, or the mechanism of Cd detoxification in the presence of Se.

Se-enriched rice, colored rice with a red seed coat, is believed to have a specific absorption capacity for Se in its roots [19]. There are currently two approaches being explored for the enrichment of Se in rice. One involves increasing the Se content in rice grains by applying exogenous inorganic Se to the leaves or into the soil [18]. The other is to produce Se-enriched offspring through the genetic combination of multiple Se-enriched parents [20]. The primary distinguishing factor between Se-enriched and non-Se-enriched rice is Se content in the rice grains, which functions of in nutrition, health care and disease prevention, and is helpful in improving Se-deficiency in humans [21]. The heavy metal Cd is better antagonized by Se-enriched rice, and the transportation of Cd from the soil, roots, and aerial parts, and especially the grains, was effectively inhibited [22]. Interestingly, it is worth noting that Se-enriched rice also employed a similar detoxification mechanism in Zn-contaminated soil [23]. However, the mechanism of action of Cd and Si in Se-enriched rice is not clear, and there are no specific recommendations for the application of Si fertilizer to Se-enriched rice in the actual Cd-contaminated soil. The purpose of our study was to identify the detoxification mechanism of Si on Cd pollution in Se-enriched rice and to determine whether the interaction of Si-Cd in Se-enriched rice followed the same trend as in non-Se-enriched rice or whether there were differences between the three Se-Cd-Si interactions. Different rice genotypes were selected for a pot experiment using basal Si fertilizer to investigate the effect of Si fertilizer at different doses on Cd accumulation in various parts of the rice plant. The findings of this study will help us select appropriate rice genotypes for Cd-contaminated areas and establish risk reduction strategies for planting crops in polluted regions.

## 2. Materials and Methods

### 2.1. Experimental Material

The Se-enriched rice maintainer line Z3055B and the non-Se-enriched rice maintainer line G46B were acquired from the Demonstration Base of International Science and Technology Cooperation of Sichuan Province, Rice Research Institute of Sichuan Agricultural University (Chengdu, China). The Se-enriched rice Z3055B [24], is a rice with a bioaccumulation effect on Se, which has been selected through years of crossbreeding and verified by the Rice Testing Centre of the Ministry of Agriculture with a cumulative Se content in polished rice of 0.064 mg/kg (GB/T 5009.93-2010), which complied with Chinese national standards for Se-enriched rice, which are 0.04–0.30 mg/kg (GB/T 22499-2008). A targeted non-Se-enriched rice that did not reach the national Se-enriched rice standard (G46B) was tested and was found to accumulate 0.023 mg/kg Se in its polished rice.

### 2.2. Nutrient Treatments Used in the Experiment

Cd and Si were obtained in a chemical compound form as silicon dioxide (SiO_2_) and cadmium chloride (CdCl_2_. 2^1^/_2_H_2_O). Both chemical elements were uniformly mixed into the test soil in the form of aqueous solutions.

### 2.3. Tested Soil

The experimental study was conducted outdoors at the Wenjiang Campus of Sichuan Agricultural University from May to September 2020, utilizing a pot-based experimental design. The test soil was naturally air-dried and crushed to remove stones, roots and other debris, and 10 kg of test soil was put into a black potting test bucket. Finally, the liquid level was maintained 5 cm above the soil surface. The basic physical and chemical properties of the soil are shown in Table 1.

### 2.4. Pot Experiment Design

The study aimed to assess the impact of distinct rice varieties on Cd bioaccumulation and Si-reduced rice’s response to Cd uptake. Soil contaminated with 1 mg/kg of Cd was used, with four levels of Si added.

The rice seeds were subjected to disinfection using a 5% sodium hypochlorite solution for a duration of 5 min, soaked for 48 h, germinated at 35 °C for 24 h, and then sown on a sand bed. When the seedlings had developed three leaves and one heart (BBCH 13), non-Se-enriched rice (G46B) and Se-enriched rice with more consistent growth state were planted in the black plastic pots (28 cm × 35 cm × 10 cm) containing 10 kg of test soil contaminated with the same Cd level, respectively, and four Si concentration treatments were set: 0 mg/kg (control), 15 mg/kg (CdSi15), 30 mg/kg (CdSi30), 60 mg/kg (CdSi60). A total of 48 pots were utilized, with 6 replicates established for each treatment. Each pot contained 4 seedlings, resulting in a total of 192 test plants.

In addition, basal fertilizer and water management were consistent with filed planting methods, and pesticides were sprayed during the period to prevent pests and diseases. After the rice matured, the rice plants were collected and washed with ultrapure water. An air-drier machine (DHG-9240B, Shen Xian Co., Ltd., Shanghai, China) was used to dry the samples at 105 °C for 15 min and then continued drying at 85 °C for 1 day to achieve a constant weight. The rice was divided into four parts: brown rice, chaff, stems and leaves, and roots. The dried material was weighed and crushed for later use. Simultaneously, the rhizosphere soil located within approximately 5 cm of the rice’s root system was collected to analyze the basic physical and chemical properties of the soil and related indicators of Cd and Se in the soil.

### 2.5. Determination Method

#### 2.5.1. Agricultural Traits

Two naturally air-dried mature panicles from each pot (a total of 12 panicles per treatment) were randomly selected to analyze the rice plant height, effective panicle number, thousand-grain weight, and grain length per pot.

#### 2.5.2. Determination of Non-Protein Thiol (NPT) Content

At the heading stage, healthy rice plants were used to obtain samples from their roots, stems, and leaves. The samples were washed, and the surface water was removed using filter paper. A 0.1 g fresh sample was weighed in a mortar, and 1 mL of extract was added for ice bath homogenization. Subsequently, 4 mL of methanol was added at room temperature, and the mixture was shaken for 10 min before being centrifuged at 10,000 r/min at 4 °C for 15 min. The resulting sample was analyzed using a Shimadzu UV—a visible spectrophotometer (T6S, Puxi Co., Ltd., Beijing, China) at an absorbance value of 412 nm, following the instructions provided by the kits from Beijing Solarbao Technology Co., Ltd. (Beijing, China).

$$NPT\;conten=\frac{14.5\times ∆A}{M}$$

#### 2.5.3. Preparation of Samples from Various Parts of the Rice

After the rice matured, the roots, leaves, and stems were washed with pure water, and then crushed into a powder form using liquid nitrogen. The resulting powder was passed through a 100-mesh sieve and stored in a −80 °C ultra-low temperature refrigerator (MDF-U3386S). The brown rice and husk were obtained by separating the rice seed part using a brown rice machine (JLG-II, Da Ji Co., Ltd., Hangzhou, China). The brown rice was further divided into polished rice, rice bran, and a small part of the embryo, using a rice mill (JNM-III, CHINA GRAIN RESERVES CORPERATION, Beijing, China). The polished rice part was extracted with tweezers, pulverized with a grinder (F160, Zhong Xing Co., Ltd., Beijing, China), passed through a 100-mesh sieve, and stored in bags in a −80 °C ultra-low temperature freezer. As a result, the rice material was divided into six parts: roots, leaves, stem, husks, brown rice, and polished rice.

The collected rice samples (roots, leaves, husks, stems, brown rice and polished rice) were put into kraft paper bags and placed in an oven (DHG-9240B, Shen Xian Co., Ltd., Shanghai, China) for drying, then they were fully crushed by a crusher (F160, Zhong Xing Co., Ltd., Beijing, China) to pass through a 100-mesh sieve. The 0.1 g powder sample was weighed and placed in a digestion vessel and digested with digestion solution (10 mL HNO_3_) in a microwave digestion apparatus (WX-600, Preekem Co., Ltd., Shanghai, China).

#### 2.5.4. Determination of Cd Content in Various Parts of the Rice

According to the method used by Farooq et al. [22], the digestion vessel was transferred to a 190 °C electric hot plate (DKQ-1800, Preekem Co., Ltd., Shanghai, China) and the solution was then allowed to be digested until it turned whitish, and the solution was then evaporated and reduced to 1 mL. Then, the 1 mL volume of the evaporated solution was diluted 3 times with 5% HCl solution, and filtered with a 0.02 μm membrane filter paper to retain a 10 mL volume of colorless and transparent liquid in a centrifuge tube which was then analyzed by an atomic absorption spectrophotometer (ICE-3300, Thermo, Waltham, MA, USA). The *Cd* content was expressed as mg/kg.

$$Cd\;content=\frac{(C-Co)\times D\times V\times 1000}{M\times 1000\times 1000}$$

*C* is the measured digestion fluid concentration of the sample (ng/mL); *C*_0_ is the concentration of the blank control group (ng/mL); *D* is the dilution factor; *M* is the sample mass; *V* is the total volume of the digestion fluid.

#### 2.5.5. Determination of Se Content in Various Parts of the Rice

An atomic fluorescence spectrophotometer was applied to determine the Se content as described previously [22]. The digestion vessel was transferred to a 120 °C electric hot plate (DKQ-1800, Preekem Co., Ltd., Shanghai, China) and subsequent rises in temperature followed, increasing by 20 °C after each 10 min interval until a final temperature of 180 °C was achieved. The digested solution was allowed to turn whitish before being evaporated, and reduced to 1 mL. Following this, the samples were diluted by 10 mL of HCl: H_2_O solution (1:1, *v*/*v*), and digested at 120 °C until a 1 mL whitish solution was generated again. The collected solution was constant to a 10 mL volume with a 5% HCl solution, prior to being analyzed using an atomic fluorescence spectrophotometer (RGF-6800, Bo Hui Co., Ltd., Beijing, China). The Se content was expressed in the units mg/kg.

$$\mathrm{Se}\;content=\frac{(C-Co)\times D\times V\times 1000}{M\times 1000\times 1000}$$

*C* is the measured *Se* concentration of the digestive solution (ng/mL); *C*_0_ is the Se concentration of the control group (ng/mL); *M* is the mass of the sample; *V* is the total volume of the digestion solution. 

#### 2.5.6. Determination of Se and Cd in Soil Samples

The 0.1 g soil sample was weighed and placed in a digestion vessel and digested with digestion solution (8 mL HNO_3_:2 mL HF) in a microwave digestion apparatus (WX-600, Preekem Co., Ltd., Shanghai, China). The digested solution was transferred to an electric hot plate (DKQ-1800, Preekem Co., Ltd., Shanghai, China), and 2 mL of HClO_4_ were added. It was then heated on an electric hot plate at 190 °C until a clear solution with a volume of less than 1 mL was separated. Then the residual 1 mL of the evaporated solution was subsequently diluted thrice with a 5% HCl solution and filtered with a 0.02 μm membrane filter paper to procure a 10 mL volume of colorless and transparent liquid in a centrifuge tube, which was analyzed utilizing an atomic absorption spectrophotometer (ICE-3300, Thermo, USA) using standard (GB-5009.93-2017).

We then added 10 mL of mixed acid solution (HCl:H_2_O) to 1 mL of transparent solution collected by the same method as above. The digestion vessels were then heated again on the electric hot plate and the temperature was increased to 120 °C for the next 8 h. The samples were carefully observed for the required clarity conditions (clean 1 mL volume). When the volume left was less than 1 mL, the solution tended to produce yellow lingering smoke. The solution was brought to a 10 mL volume with a 5% HCl solution, prior to analysis using an atomic fluorescence spectrophotometer (RGF-6800, Bo Hui Co., Ltd., Beijing, China) using standard (GB-5009.15-201).

#### 2.5.7. Inorganic Se Measurement Method

According to the local standard of Jiangxi Province of China (DB36/T 1243-2020), about 2.5 g of sample was placed in a 50 mL graduated test tube with a stopper and 20 mL of hydrochloric acid solution was added. The tube was placed in a 70 °C constant-temperature water bath, shaken at between 100 r/min to 200 r/min for 2 h, then cooled to room temperature. After being filtered through absorbent cotton, the cotton ball was rinsed with a small amount (less than 15 mL) of hydrochloric acid solution and the filtrate was collected to make the volume 50 mL. The filtrate was extracted with 5 mL of cyclohexane, and 25 mL of water phase was collected in a 50 mL graduated test tube with a stopper and heated in a boiling water bath for 20 min, then cooled to room temperature. Then, 2.5 mL of potassium ferricyanide solution, three drops of n-octanol, and water were added to make the volume 50 mL, and finally measured using an atomic fluorescence spectrophotometer to measure the inorganic Se.

#### 2.5.8. Organic Se Measurement Method

The subtraction method was used: Organic Se = Total Se − Inorganic Se.

### 2.6. Data Analysis

To detect the elements, samples were obtained from two plants, and three biological repeats were utilized for each part (roots, stems, leaves, husks, brown rice and polished rice). The assembled information underwent ANOVA through IBM SPSS Statistics v.26.0, and the resultant data were scrutinized using GraphPad Prism 7.0 for the construction of graphical representations.

## 3. Results

### 3.1. Agronomic Traits

**Figure 1:**
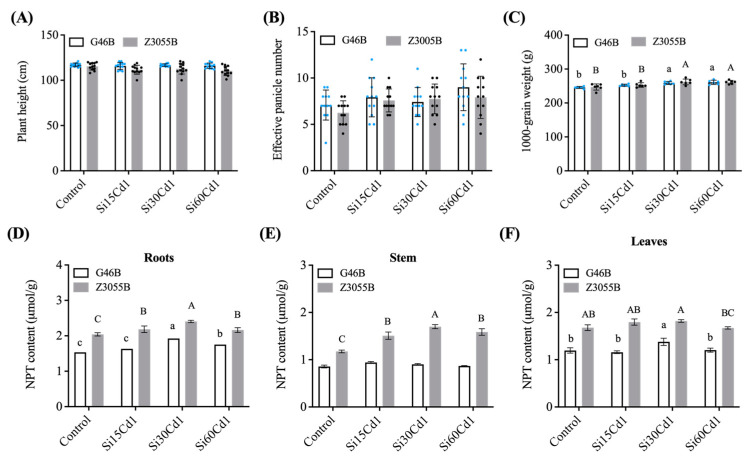
Agronomic traits and non-protein thiol content (NPT) of roots, stem and leaves of two different genotypes of rice. (**A**) Plant height of non-Se-enriched rice G46B and Se-enriched rice Z3055B. Data are mean ± SEM, *n* = 12 samples. (**B**) Effective number of panicles of non-Se-enriched rice G46B and Se-enriched rice Z3055B. Data are mean ± SEM, *n* = 12 samples. (**C**) 1000-grain weight of non-Se-enriched rice G46B and Se-enriched rice Z3055B. Data are mean ± SEM, *n* = 6 samples. (**D**–**F**) NPT content in roots, stem and leaves of non-Se-enriched rice G46B and Se-enriched rice Z3055B, respectively. Data are mean ± SEM, *n* = 3 samples. Lowercase letters indicate significant differences in non-Se-enriched rice G46B (*p* < 0.05); Capital letters indicate significant differences in Se-enriched rice Z3055B (*p* < 0.05).

In order to shed light on the growth patterns of Se-enriched rice Z3055B and non-Se-enriched rice G46B in the presence of Cd pollution, we conducted an analysis of several key agronomic traits. This investigation aimed to provide insight into the impact of Cd pollution on the growth dynamics of these two rice varieties. Field observations showed that both genotypes had a lower seed setting rate and a higher number of empty husks under Cd stress. After the addition of Si fertilizer, plant growth was improved profoundly, with a marked increase in thousand-grain weight and a reduction in the incidence of shattered grains and empty husks. Interestingly, no statistically significant distinctions were observed in the plant height (Figure 1A) or effective panicle number per plant (Figure 1B) between Se-enriched and non-Se-enriched genotypes across various Si treatments. A noteworthy aspect is that as the level of Si application increased, there was a clear trend of increasing 1000-grain weight, with the most significant increase observed at 30 mg/kg and 60 mg/kg treatments (Figure 1C).

### 3.2. NPT Content

The results demonstrated that under Cd stress, basal fertilizer application of Si was beneficial in the combination of rice NPT and heavy metal ions, offering a detoxification mechanism against Cd stress. The contents of NPT in roots, stems and leaves of non-Se-enriched rice G46B and Se-enriched rice Z3055B were presented in Figure 1D–F. Under Cd pollution, Si treatment significantly enhanced the bioavailability of NPT in Se-enriched rice roots, stems, and leaves, leading to a significant increase in NPT content in each part. However, this biofortification effect was not as pronounced in non-Se-enriched rice. 

As depicted in Table 2, the content of NPT was significantly impacted by both the level of Si treatment and the type of genotype, with a notable interaction effect. The treatments exhibited significant variations, and different Si levels were found to result in considerable differences in the NPT contents of both roots and leaves when compared to the control. Moreover, Se-enriched rice strain Z3055B demonstrated significantly higher NPT content in roots, stems, and leaves compared to the non-Se-enriched rice strain G46B.

It is worth emphasizing that the NPT content in the roots, stems, and leaves of Z3055B was significantly higher than that of G46B, thereby suggesting that Se-enriched rice has a superior ability to counteract Cd and provide more efficient detoxification against this heavy metal. Notably, the highest threshold of NPT content in Se-enriched rice roots, stems, and leaves was observed under T30 treatment, reaching an impressive 2.41 μmol/g, 1.70 μmol/g and 1.82 μmol/g, respectively (Figure 1).

### 3.3. Cd Concentration in Different Parts of Plants of Two Different Genotypes of Rice

**Figure 2:**
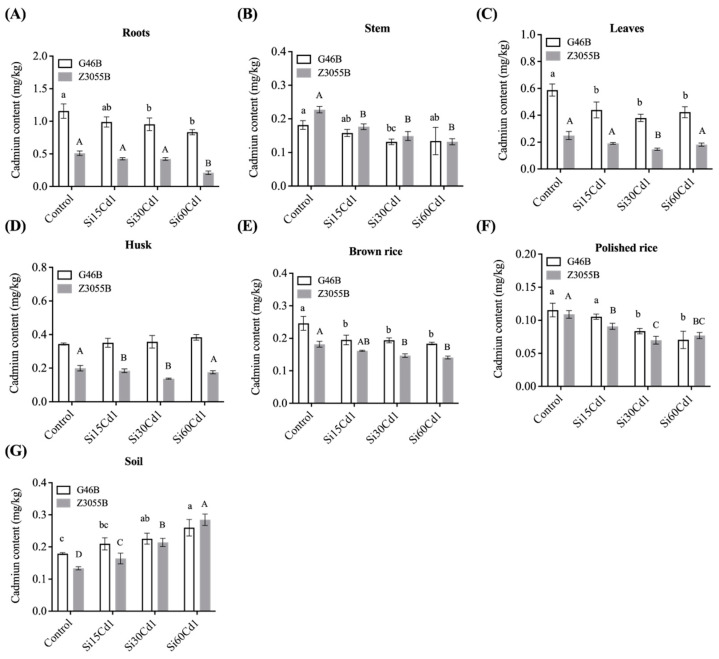
Cadmium content in various parts of soil and two different genotypes of rice. (**A**–**G**) Cadmium content in roots, stem, leaves, husk, brown rice, polished rice and soil of non-Se-enriched rice G46B and Se-enriched rice Z3055B, respectively. Data are mean ± SEM, *n* = 3 samples. Lowercase letters indicate significant differences in non-Se-enriched rice G46B (*p* < 0.05); Capital letters indicate significant differences in Se-enriched rice Z3055B (*p* < 0.05).

The impact of basal fertilizer application of Si on Cd absorption and accumulation in different parts of the rice was investigated in this study (Figure 2). The Cd accumulation trend of different rice genotypes under different Si treatments was basically maintained under the heavy metal Cd stress.

The enrichment of Cd in the above-ground and underground parts of the rice plant was significantly affected by the Si treatment level and by genotype, with significant differences observed between different parts of the plant (Table 3). Among them, the interaction effect between Si treatment level and the different genotypes varied, depending on the plant part, with only the enrichment of Cd in stem and husk showing a significant interaction effect. However, within the scope of this study, Cd content in all parts of the rice significantly decreased with increasing levels of Si. Interestingly, Cd content was significantly lower in all parts of Se-enriched rice Z3055B than in non-Se-enriched rice G46B. 

### 3.4. Se Concentration in Different Parts of Two Different Genotypes of Rice

**Figure 3:**
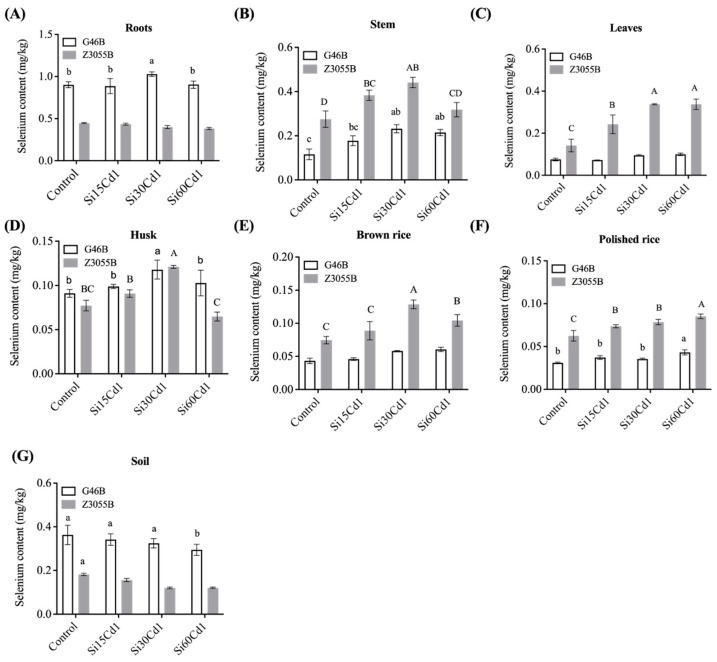
Selenium content in various parts of two different genotypes of rice under different levels of Si fertilizer. (**A**–**G**) Selenium content in roots, stem, leaves, husks, brown rice, polished rice and soil of non-Se-enriched rice G46B and Se-enriched rice Z3055B, respectively. Data are mean ± SEM, *n* = 3 samples. Lowercase letters indicate significant differences in non-Se-enriched rice G46B (*p* < 0.05); Capital letters indicate significant differences in Se-enriched rice Z3055B (*p* < 0.05).

The uptake and accumulation of Se in rice plants was significantly influenced by Si fertilization and genotype, as indicated by the significant interaction effect (Table 4) and demonstrated in Figure 3. Non-Se-enriched rice (G46B) showed a root > husk > leaves > stem > brown rice > polished rice trend for Se enrichment, with significant increases in Se content in each part under 30 mg/kg Si treatment. However, Se accumulation in the polished rice of G46B did not meet the Se-enriched standard, as the Se was mainly concentrated in the roots and was not transported to the grains. On the other hand, Se-enriched rice efficiently absorbed Se from the soil and transferred it to the grains, resulting in polished rice that complied with the Chinese national standard for Se-enriched rice. The optimal Si treatment concentration for increasing Se and decreasing Cd in Se-enriched rice was found to be 30 mg/kg, with significant increases in Se content in each part, compared to the control. The maximum total Se content in the polished rice reached twice the maximum value of non-Se-enriched polished rice under 60 mg/kg Si treatment (Figure 3F).

### 3.5. Distribution of Inorganic Se and Organic Se in Brown Rice of Two Genotypes

**Figure 4:**
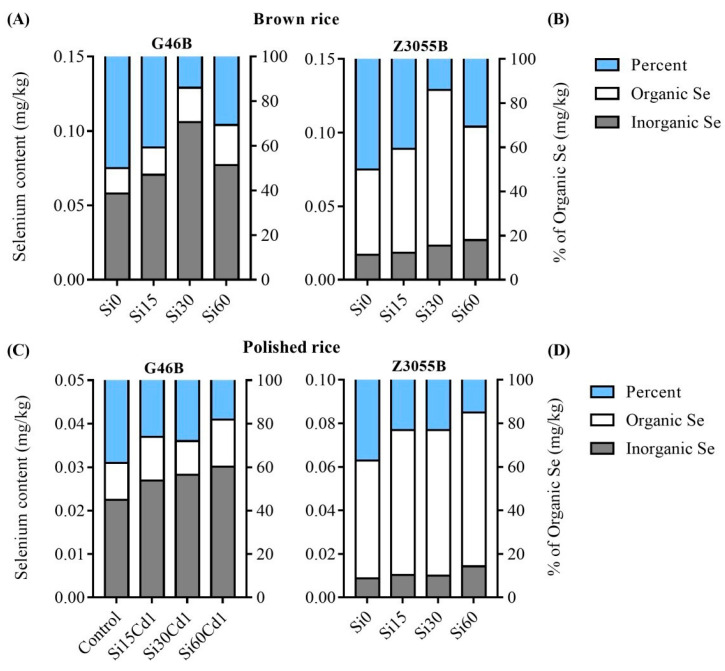
Inorganic selenium and organic selenium content in brown and polished rice of non-Se-enriched rice G46B and Se-enriched rice Z3055B. (**A**) Inorganic Se and organic Se content in brown rice of non-Se-enriched rice G46B. (**B**) Inorganic Se and organic Se content in brown rice of Se-enriched rice Z3055B. (**C**) Inorganic Se and organic Se content in polished rice of non-Se-enriched rice G46B. (**D**) Inorganic Se and organic Se content in polished rice of Se-enriched rice Z3055B. Data are mean ± SEM, *n* = 3 samples.

The levels of inorganic Se and organic Se in brown rice of both genotypes were significantly influenced by Si treatment level and cultivar type, as well as their interaction (Table 4). In non-Se-enriched G46B, the content of both Se forms increased significantly with higher Si fertilizer concentrations, peaking in the Cd1Si60 treatment group, with 0.021 mg/kg and 0.040 mg/kg for inorganic Se and organic Se, respectively. Moreover, the proportion of organic Se reached a maximum of 66.26% under the Si treatment of 30 mg/kg (Figure 4A). For Se-enriched Z3055B, the organic Se content in brown rice significantly increased with the increased Si levels, with a range between 0.058 mg/kg and 0.11 mg/kg. The optimal concentration for organic Se was observed in the Cd1Si30 group, with a content of 0.11 mg/kg and a proportion of 82.0% (Figure 4B).

### 3.6. Distribution of Inorganic Se and Organic Se in Polished Rice of Two Genotypes of Rice

The results of the study demonstrate that both the level of Si treatment and the genotype have a significant impact on the enrichment of organic Se in polished rice, with a significant interaction effect. However, no significant difference was observed in the transport and storage of inorganic Se (Table 4). The content of both inorganic Se and organic Se in non-Se-enriched and Se-enriched polished rice increased significantly with increasing Si concentration in the base fertilizer (Figure 4). Notably, the content of inorganic Se and organic Se in both types of rice was maximized at the Si application level of 60 mg/kg.

Interestingly, at the Si treatment of 30 mg/kg, the maximum percentage of organic Se was achieved, with values of 78.4% (G46B) and 87.1% (Z3055B), respectively. This finding suggests that 30 mg/kg was the optimal Si concentration treatment for achieving the highest organic Se content in polished rice, regardless of its Se-enrichment status (Figure 4C,D).

## 4. Discussion

Cd was not a nutrient; in fact, most animals and plants were poisoned by excessive accumulations of Cd [25,26]. The toxicity of Cd on plants is primarily due to the reduction of photosynthetic efficiency that results from stomatal occlusion, suppression of pigment biosynthesis, deactivation of the enzymes associated with CO_2_ fixation, and damage of to the photosynthetic mechanisms—particularly, the two light systems and daylighting complex II [27]. Some studies [25,28] also believed the inhibitory effects of Cd on growth could be attributed to the suppression of both cell division and cell elongation, which was mainly caused by the irreversible hindrance of the proton pump responsible for facilitating these processes.

Changes in above-ground biomass were found to affect the variation in Cd stress adaptation [29], as the minimal decrease in above-ground biomass was interpreted as a sign of elevated stress tolerance in plants. Several studies have proposed that transcription factors and metal binding proteins were replaced by an excessive accumulation of Cd, which disrupted the homeostasis of essential metals in plants [30], causing a range of physiological disturbances and inhibiting rice growth [31], which was consistent with our research results. The growth of two rice varieties was significantly enhanced and the impact of medium-dose Cd stress was effectively alleviated with the addition of exogenous Si at a level of 30 mg/kg. The thousand-grain weight was significantly increased, and the occurrence of shattered grains and empty husks was reduced. This was because the cellulose-related silica gel layer in the plant epidermal cell wall was thickened by the Si, the loss of cell water was reduced, plant transpiration was weakened, and the internal water stress of the plant was reduced, ultimately increasing the plant biomass and rice yield [32,33].

NPT, composed of substances containing sulfhydryl groups, plays a crucial part in the metal tolerance mechanism of plants [34]. Its main function is to complex metal ions and prevent heavy metal toxicity [35]. The results obtained are similar to the findings of current research. The exposure of two rice varieties to heavy metal Cd stress after the addition of exogenous Si resulted in a notable rise in NPT content throughout the roots, stems, and leaves. This reveals that the supply of exogenous Si can enhance the content of non-protein sulfhydryl substances in rice to some extent. Previous research [36] concluded that a quantity of exogenous Si noticeably enriched the NPT content in each part of the plant and the toxicity of Cd was reduced, which agrees with our research results. The reason for the observed increase and subsequent decrease in NPT content may be attributed to the stimulation of the redox system of rice cells by with the supplementation of low levels of Si, while the downward trend at high Si levels may be due to some limitations to plant resistance to heavy metals [37]. Heavy metal stress in rice was primarily concentrated in the parts of the plant with strong physiological activity, and specifically in the roots and leaves, which exhibited the most significant response. 

The enrichment of heavy metals in plant roots and above-ground parts was one of the prominent indicators for assessing the capacity for resistance to heavy metal pollution [38]. The amount of Cd in plant tissues was in the order of roots > leaves > husk > brown rice > stem > polished rice. Heavy metal Cd accumulates more in organs with vigorous metabolism such as roots, but less in nutrient storage organs such as stems and leaves [39]. This natural response by plants to heavy metal stress may serve as a form of self-protection [40]. The incorporation of Si resulted in a remarkable decrease in the level of Cd in each component of the rice plant, implying that Si-mediated Cd toxicity mitigation may entail the retention of Cd in the roots and the abatement of Cd translocation from the roots to the shoots [32]. The flow of the apoplast from the roots of the rice plants to the above-ground transport pathway was limited by the co-precipitation of Si and Cd formed in rice epidermal cells, which stabilized Cd in the plasmodesmata of the stalk. Cd and Si exhibited a competitive effect in the transportation process, which impeded the movement of Cd from the rice roots beneath the soil to the aerial parts above ground [41]. The mechanism behind Si’s inhibition of Cd accumulation from roots to brown rice lied in the transport from roots to stalks. As the translocation of Cd from roots to stalks was alleviated, the accumulation of Si in the stalks may also have decreased. Previous studies have demonstrated that Si forms complexes with cell wall hemicellulose. These Si complexes carried negative charges that enhanced binding to Cd, thereby inhibiting the transport of Cd [42].

Furthermore, Se-enriched rice variety Z3055B exhibited superior resistance to the heavy metal Cd compared to the non-Se-enriched rice variety G46B. Similar studies have indicated that heavy metals were better antagonized by Se-enriched rice, which altered the deposition in the aerial portions of plants. The possible reason was that the mechanism of Se regulating heavy metals was activated, as prior research observed that exposure to Se diminished the uptake and toxicity of Cd in plants such as winter wheat [43], Chinese cabbage [38] and sunflowers [25]. Se was beneficial to plants at low doses, which was used to alleviate abiotic and biotic stress in the organism [26,44]. These findings indicated that the possible mechanism by which Se reduced the bioaccumulation of heavy metals in rice was through the reduction of root translocation, the synthesis of Cd chelates within the cytoplasm of root cells and their subsequent sequestration into vacuoles to impede the translocation of Cd from plasmodesmata to xylem [45,46]. Filek et al. [47] reported that the decline in root absorption of Cd was partially attributed to the co-transportation of Cd and Se via the same protein carrier, which restrained the metabolism of Cd in the active membrane. Our study showed that the use of Si fertilizer effectively alleviated the stress of Cd on Se-enriched rice, although the effect was not significant for non-Se-enriched rice. The primary cause could be the presence of certain factors existing in the roots. We postulated that the Si-mediated abatement of Cd absorption in rice may have a positive impact on Se uptake. Given that Se and Cd share a common protein carrier, the competition between them could impede the upward transport of Cd in rice. This explains the observed variability in Se absorption across different rice varieties, owing to inherent differences in their genotypes. Eventually, we observed diametrically opposite trends in the absorption of Cd and Se by the roots of Se-enriched and non-Se-enriched rice. To fully elucidate the underlying mechanisms that regulate the growth and development of Cd and Si in Se-enriched rice, further molecular investigations are warranted to account for any unknown variables that may be involved.

Our current research has revealed an intriguing discovery—the Se content in each part of Se-enriched rice was considerably greater than that of non-Se-enriched rice, with the exception of the roots (Figure 3). Surprisingly, in the absence of silicon fertilizer treatment, Se-enriched polished rice was still able to achieve the Se enrichment standard, while the roots of non-Se-enriched rice exhibited an even greater concentration of Se than that of the Se-enriched rice. The outcomes of Farooq et al. [19] were in concurrence with this study. However, another study suggested that Se-enriched rice varieties absorbed more Se due to their superior root structure [48], contrary to our findings. We propose that the reason why Se absorbed by non-Se-enriched rice accumulated in the roots was not because of a limiting factor in the root structure, but rather a lack of efficient Se transporters. In contrast, Se-enriched rice possessed more transporters to meet Se transport requirements. The expression levels of *Sutr1;2*, *OsPT2* and *OsNIP2;1* genes were higher in Se-enriched rice, compared to non-Se-enriched rice treated with selenate or selenite. Se assimilation in Arabidopsis was regulated by *Sutr1;2,* and Se uptake in its roots was enhanced with an overexpression of *OsPT2* in rice [44]. Farooq et al. [22] showed that Se-enriched rice was more sensitive to Se in the soil, which facilitated the translocation of Se from the below-ground parts into the grain, with storage in brown rice in the form of organic Se (Figure 4B) and polished rice better than 80% (Figure 4D). In addition, *NRT1.1B,* a constituent of the rice peptide transporter (PTR) family, was thought to enhance Se accumulation in grains by promoting the transport of selenomethionine (SeMet). This provides valuable insight into the development of new breeding strategies for producing Se-enriched rice varieties [49]. Non-Se-enriched rice was able to absorb a limited amount of Se from the soil and transport it into the grain; organic Se accounts for 60% of the enrichment in both brown rice (Figure 4A) and polished rice (Figure 4C), while the transport of Se from the glume to the embryo and endosperm was hindered due to inadequate capacity of the inner milk cells to donate inorganic Se, as suggested by previous research [50]. It remains unclear whether there are any synergistic or related effects on the assimilation of silicon (Si) and Se for different rice varieties. In our research, we observed a significant correlation between Si treatment and Se content in all parts of the rice (Table 4), which supports the idea that Si is beneficial for the enrichment and transport of Se in rice.

In the current working model, the chelation and compartmentalization of Si and Cd in rice diminished Cd absorption and transferred it to the shoot in rice. However, we have noticed varying trends in Se-enriched rice. Our findings supported the idea that Se-enriched rice was more resistant to heavy metal Cd pollution than non-Se-enriched rice, and the ability to use Si fertilizer to alleviate Cd pollution is more pronounced in Se-enriched rice. It is possible that Se-enriched rice possesses its own detoxification system for heavy metal Cd. Therefore, specific management strategies should be adopted for rice varieties in a Cd-contaminated environment, based on their individual conditions. In the future, it is hoped that relevant genetic factors can be identified and verified to better understand the underlying mechanisms.

## 5. Conclusions

This study demonstrated that Se-enriched rice exhibited a different pattern of cadmium enrichment compared to regular rice, suggesting that Se-enriched rice possesses a stronger ability to resist cadmium pollution and can better alleviate its effects through the addition of silicon fertilizer. This highlights the need for different management practices to be employed for rice cultivation in environments with high levels of Cd pollution. These findings will aid in selecting the optimal rice genotypes and management techniques for cultivation in Cd-contaminated regions, and will provide a foundation for developing strategies to reduce the pollution risks associated with crop production.

## Figures and Tables

**Table 1:** Basic physical and chemical properties of the tested soil.

Index	Content	Method
pH	5.93 ± 0.066	NY/T 1377-2007
Organic matter (g/kg of soil)	28.89 ± 2.233	NY/T 1121.6-2006
Total nitrogen (g/kg of soil)	0.191 ± 0.012	HJ 717-2014
Total phosphorus (g/kg of soil)	1.49 ± 0.015	NY/T 88-1988
Total potassium (g/kg of soil)	10.3 ± 0.931	NY/T 87-1988
Total selenium (mg/kg of soil)	0.22 ± 0.0298	GB 5009.93-2017
Total Cadmium (mg/kg of soil)	0.085 ± 0.008	GB/T17141-1997

Data are mean ± SD, *n* = 3 samples.

**Table 2:** Agronomic traits and non-protein thiol content (NPT) of roots, stem and leaves of two different genotypes of rice.

Interaction Effects		Plant Height (cm)	Effective Panicle Number	1000-Grain Weight (g)	NPT Content		
					Roots (µmol/g)	Stem (µmol/g)	Leaves (µmol/g)
Treatment (T)	Control	116.157	6.667b	246.379b	1.788c	1.016c	1.437b
	Si15Cd1	113.735	7.75ab	252.697b	1.909b	1.225b	1.478b
	Si30Cd1	114.75	7.578ab	260.673a	2.165a	1.301a	1.598a
	Si60Cd1	113.25	8.458a	261.133a	1.957b	1.227b	1.438b
	SEm±	0.828	0.374	1.776	0.022	0.018	0.021
Genotypes	G46B	116.417	7.854a	254.565	1.71b	0.892b	1.234b
	Z3055B	112.542	7.375b	255.867	2.199a	1.493a	1.742a
	SEm±	0.585	0.264	1.256	0.016	0.013	0.015
Interaction	T	ns	*	**	**	**	**
	G	**	ns	ns	**	**	**
	T × G	ns	ns	ns	ns	**	*

Note: T represents different silicon treatments; G represents different genotypes; T × G represents the interaction between treatments and varieties; values within a column followed by different letters are significantly different at *p* < 0.05; *, ** Significantly different at 0.05 and 0.01 probability levels, ns denote non-significance (*p* > 0.05), respectively.

**Table 3:** Cadmium content in various parts of two different genotypes of rice under different levels of Si fertilizer.

Interaction Effects		Cadmium Concentration (mg/kg)						
		Soil	Roots	Stem	Leaves	Husk	Brown Rice	Polished Rice
Treatment (T)	Control	0.156d	0.835a	0.205a	0.419a	0.272a	0.214a	0.112a
	Si15Cd1	0.187c	0.708b	0.167b	0.316b	0.268ab	0.178bc	0.098
	Si30Cd1	0.22b	0.689b	0.140c	0.264bc	0.247ab	0.170bc	0.77c
	Si60Cd1	0.272a	0.523c	0.133c	0.303c	0.280b	0.162c	0.077c
	SEm±	0.007	0.026	0.007	0.014	0.008	0.04	0.003
Genotypes (G)	G46B	0.219a	0.984a	0.151b	0.458a	0.359a	0.205a	0.097a
	Z3055B	0.199b	0.393b	0.171a	0.193b	0.174b	0.158b	0.085b
	SEm±	0.018	0.018	0.01	0.01	0.06	0.03	0.002
	T	**	**	**	**	ns	**	**
	G	**	*	**	**	**	**	*
Interaction	T × G	**	ns	*	ns	*	ns	ns

Note: T represents different silicon treatments; G represents different genotypes; T × G represents the interaction between treatments and varieties; values within a column followed by different letters are significantly different at *p* < 0.05; *, ** Significantly different at 0.05 and 0.01 probability levels, ns denote non-significance (*p* > 0.05), respectively.

**Table 4:** Selenium content in various parts of two different genotypes of rice under different levels of Si fertilizer.

Interaction Effects		Selenium Content (mg/kg)							Organic Se Content (mg/kg)		Inorganic Se Content (mg/kg)	
		Soil	Roots	Stem	Leaves	Husk	Brown Rice	Polished Rice	Brown Rice	Polished Rice	Brown Rice	Polished Rice
Treatment (T)	Control	0.272a	0.674ab	0.196c	0.109c	0.059c	0.045c	0.045c	0.042c	0.08c	0.017c	0.009
	Si15Cd1	0.249ab	0.66ab	0.281b	0.157b	0.067c	0.055b	0.055b	0.05bc	0.045b	0.018c	0.01
	Si30Cd1	0.222bc	0.714a	0.337a	0.217a	0.093a	0.057b	0.057b	0.072a	0.048b	0.021b	0.009
	Si60Cd1	0.208d	0.644b	0.266b	0.219a	0.082b	0.064a	0.064a	0.058b	0.054a	0.024a	0.01
	SEm±	0.009	0.016	0.01	0.009	0.003	0.00	0.00	0.033	0.001	0.001	0.001
Genotypes (G)	G46B	0.33a	0.93a	0.185b	0.086b	0.052b	0.037b	0.037b	0.027b	0.027b	0.019b	0.009
	Z3055B	0.145b	0.416b	0.355a	0.265a	0.099	0.075a	0.075a	0.054a	0.065a	0.021a	0.01
	SEm±	0.006	0.011	0.007	0.006	0.002	0.001	0.001	0.002	0.001	0.001	0.001
	T	**	**	**	**	**	**	**	**	**	**	ns
	G	**	**	**	**	**	**	**	**	**	**	ns
Interaction	T × G	ns	**	**	**	**	**	*	*	*	*	ns

Note: T represents different silicon treatments; G represents different genotypes; T × G represents the interaction between treatments and varieties; values within a column followed by different letters are significantly different at *p* < 0.05; *, ** Significantly different at 0.05 and 0.01 probability levels, ns denote non-significance (*p* > 0.05), respectively.

## Data Availability

No new data were created or analyzed in this study. Data sharing is not applicable to this article.
